# Diagnosis of sarcopenia on thoracic computed tomography and its association with postoperative survival after anatomic lung cancer resection

**DOI:** 10.1038/s41598-023-45583-5

**Published:** 2023-10-27

**Authors:** Simone Kaltenhauser, Christoph Niessen, Florian Zeman, Christian Stroszczynski, Niels Zorger, Jirka Grosse, Christian Großer, Hans-Stefan Hofmann, Tobias Robold

**Affiliations:** 1 Department of Thoracic Surgery, University Hospital Regensburg, Franz-Josef-Strauss-Allee 11, 93053 Regensburg, Germany; 2 Department of Radiology, Caritas-Krankenhaus St Josef, Regensburg, Germany; 3 Center of Clinical Studies, University Hospital Regensburg, Regensburg, Germany; 4 Department of Radiology, University Hospital Regensburg, Regensburg, Germany; 5 Department of Radiology, Hospital Barmherzige Brüder Regensburg, Regensburg, Germany; 6 Department of Nuclear Medicine, University Hospital Regensburg, Regensburg, Germany; 7 Department of Thoracic Surgery, Hospital Barmherzige Brüder Regensburg, Regensburg, Germany

**Keywords:** Lung cancer, Surgical oncology, Risk factors, Computed tomography, Prognosis

## Abstract

Computer tomography-derived skeletal muscle index normalized for height in conjunction with muscle density enables single modality-based sarcopenia assessment that accounts for all diagnostic criteria and cutoff recommendations as per the widely accepted European consensus. Yet, the standard approach to quantify skeletal musculature at the third lumbar vertebra is limited for certain patient groups, such as lung cancer patients who receive chest CT for tumor staging that does not encompass this lumbar level. As an alternative, this retrospective study assessed sarcopenia in lung cancer patients treated with curative intent at the tenth thoracic vertebral level using appropriate cutoffs. We showed that skeletal muscle index and radiation attenuation at level T10 correlate well with those at level L3 (Pearson’s R = 0.82 and 0.66, p < 0.001). During a median follow-up period of 55.7 months, sarcopenia was independently associated with worse overall (hazard ratio (HR) = 2.11, 95%-confidence interval (95%-CI) = 1.38–3.23, p < 0.001) and cancer-specific survival (HR = 2.00, 95%-CI = 1.19–3.36, p = 0.009) of lung cancer patients following anatomic resection. This study highlights feasibility to diagnose sarcopenia solely by thoracic CT in accordance with the European consensus recommendations. The straightforward methodology offers easy translation into routine clinical care and potential to improve preoperative risk stratification of lung cancer patients scheduled for surgery.

## Introduction

Sarcopenia (Greek sarx 'flesh' and penia 'poverty') is commonly described as a progressive and generalized disorder of skeletal muscle^1^. Beyond ageing^1,2^, many factors have been identified to contribute to the sarcopenic phenotype, such as malnutrition, inactivity and chronic diseases like chronic obstructive pulmonary disease, diabetes mellitus and cancer^3^. Within different cancer types and stages including lung cancer, sarcopenia was shown to be associated with poorer survival^4^.

To date, no international consensus on sarcopenia has been achieved^3^. And so far, different approaches have been used to quantify sarcopenia^1,5^. The most widely used definition and diagnostic criteria for sarcopenia have been established by the European Working Group on Sarcopenia in Older People (EWGSOP)^1^ and supported by the Asian Working group on Sarcopenia^2^. They have been endorsed for clinical practice and research by various international scientific societies^3^. Accordingly, sarcopenia can be determined by muscle strength, quantity, quality, and physical performance^1,2^. Lumbar third vertebral level (L3) imaging by computed tomography (CT) is considered gold standard for non-invasive muscle assessment^1^. Muscle quantity can be measured by cross-sectional muscle area (SMA) on axial CT imaging and converted into a height-adjusted index, skeletal muscle index (SMI), similar to body mass index (BMI). CT-derived skeletal muscle radiation attenuation (SMRA) is a measure of muscle quality^1,6,7^ which is associated with function^7–10^, strength^7,11,12^ and physical performance^13^. Hence, SMA or SMI in combination with SMRA may enable complete assessment of sarcopenia, solely by CT, if respective cutoff values are available^13^. The general recommendation is to set the cutoff for sarcopenia-related measurements two standard deviations (SD) below the mean of a young and healthy reference population^1^. For lumbar level L3 many reference values have been reported^14,15^. However, as part of the standard of care staging, lung cancer patients usually undergo CT of the chest and upper abdomen that does not include L3^16^. European consensus based CT-cutoff values derived from a healthy cohort of potential kidney donors have been presented for thoracic spine levels^13^ that, to our knowledge, have not yet been tested for clinical outcomes.

We performed a novel approach using SMI in conjunction with SMRA to provide an assessment of sarcopenia solely by thoracic CT that meets the European consensus definition and diagnostic criteria^1^ and analyzed its relevance for preoperative risk stratification for long-term survival of lung cancer patients after anatomic resection.

## Patients and Methods

### Data acquisition

**Figure 1:**
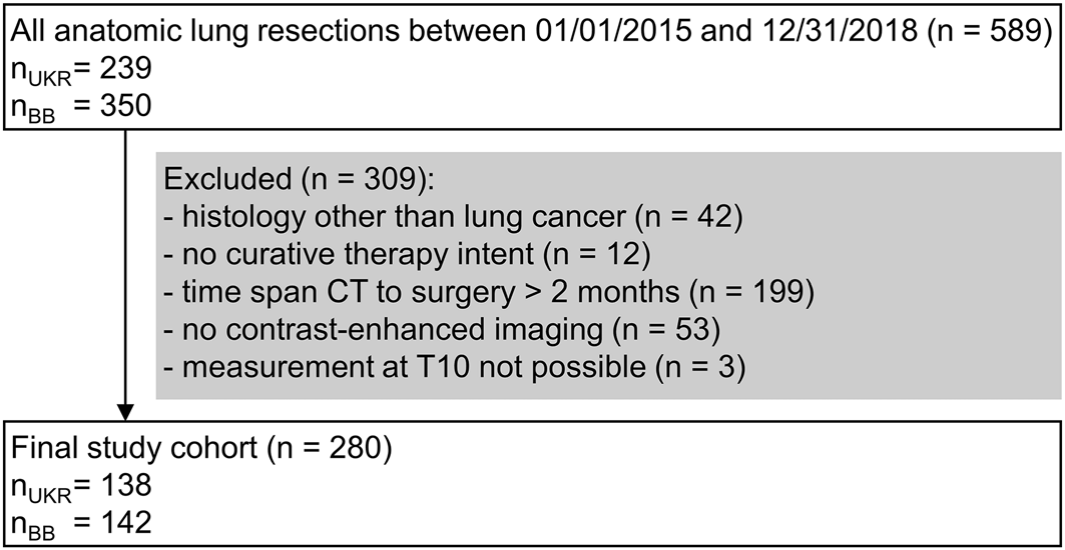
Patient inclusion/exclusion flowchart. *UKR* University Hospital Regensburg, *BB* Hospital Barmherzige Brüder Regensburg.

Data was acquired at two tertiary hospitals, the University Hospital Regensburg and Hospital Barmherzige Brüder Regensburg. For both institutions, the study protocol and ethical clearance were approved by the Institutional Review Board (IRB) of the University Regensburg (Study code 20-2045-101, approved on October 14, 2020). All methods were carried out in accordance with institutional guidelines and regulations. Because pre-existing patient information and CT scans were used retrospectively, the need for informed consent was waived for both hospitals by the IRB of the University Regensburg. At both tertiary institutions, we retrospectively identified 589 patients who underwent anatomic pulmonary resection (segmentectomy, (bi-)lobectomy or pneumonectomy) in the departments of thoracic surgery between 2015 and 2018 (Fig. 1). Inclusion required pathologically confirmed diagnosis of lung cancer and treatment with curative intent. Preoperative contrast-enhanced computed tomography examinations of the thorax and abdomen were included if they were obtained within 60 days before surgery with complete visualization of skeletal muscle at the level of the tenth thoracic vertebral body (T10). Clinical data were extracted from the most recent patient records before surgery. Comorbidities were assessed by the Charlson Comorbidity Index (CCI) based on the information of the preoperative patient report^17,18^. Since 2017, the staging of lung cancer has been performed according to the eighth edition of the Tumor, Nodes, and Metastasis (TNM) staging system of malignant tumors. Thus, patients who had surgery before 01/01/2017 were classified according to the criteria of the eighth edition based on the seventh edition TNM descriptors^19,20^. From preoperative pulmonary function testing, forced expiratory volume in one second (FEV1), diffusion capacity for carbon monoxide (DLCO), and vital capacity (VC) were retrieved. Primary outcomes were overall and cancer-specific survival. Cancer-specific decease was determined by death from the index lung cancer. Survival time was calculated from the day of resection. Vital status and date of last follow-up were retrieved from medical records, death certificates, registration offices, and the Clinical Cancer Registry of the Tumor Center–Institute for Quality Management and Health Services Research, University of Regensburg. If no death record was found, survival was censored at date of last visit. The follow-up period ended on 05/31/2022.

### Semi-automated CT-based muscle measurement

The most recent preoperative CT image was accessed with free-ware 64-bit DICOM viewer Horos™ (Horos Project, Geneva, Switzerland, version 4.0). Patients underwent routine preoperative CT imaging at a tube voltage of 100 kVp and with contrast injection yielding 3 to 5 mm thick multiplanar reconstructions in the axial plane.

Measurements were performed at the level of the fifth, eighth and tenth thoracic vertebral bodies (T5, 8, 10) and at the third level of the lumbar vertebral body (L3). 

Lumbar third vertebra imaging by CT is considered gold standard for non-invasive skeletal muscle and sarcopenia assessment^1^. T5 serves as an anatomic landmark for the aortic arch and has been previously used to quantify skeletal muscle^21^. Nevertheless, complete visualization is more frequently available further caudal^22,23^. T8 was shown to correlate well with T5^23^. More importantly, T10 is the most cranial level for which cutoff values have been established^13^. The selection of vertebral levels also allowed us to analyze the correlation of skeletal muscle measurements of the upper, middle, and lower thoracic and lumbar spine.

We measured on two consecutive CT slices where both procc. tranversi of the thoracic spine or procc. costales of the lumbar spine were visible and computed the mean of both measurements for further analysis. The distinction between different tissue components is based on Hounsfield units (HU). Using the “Grow Region (2D/3D Segmentation)” application within the Region-of-Interest-tool, a preset threshold range of −30 to 150 HU^7^ was used to automatically quantify cross-sectional skeletal muscle area and the respective mean skeletal muscle radiation attenuation within the area. If necessary, subsequent manual adjustment was performed by a primary analyst with consensus reading of a board-certified radiologist with more than 10 years of experience. 

### Sarcopenia assessment

According to the European consensus guidelines, sarcopenia is confirmed by the presence of low muscle quantity or quality in combination with low muscle strength. Poor physical performance indicates severe sarcopenia^1^. Cross-sectional muscle area (cm^2^) was normalized for height (m) squared and reported as skeletal muscle index (cm^2^/m^2^). We diagnosed sarcopenia by presence of low SMI in combination with low SMRA which were defined as SMI of < 28.8 cm^2^/m^2^ for men and < 20.4 cm^2^/m^2^ for women and SMRA < 32.4 HU for men and < 26.5 HU for women^13^, respectively.

### Statistical methods

Statistical analyses were performed with R version 4.2.0^24^ and the package ggplot2^25^ for data visualization. The significance-level was set at p < 0.05 for hypothesis testing and confidence intervals (CI) unless otherwise indicated. We present categorical variables as count [percentage (%)] and continuous variables as mean [standard deviation (SD)] or median [interquartile range (Q1–Q3)], as appropriate. Differences between groups were analyzed with the chi-square test of independence or Fisher exact test (if the expected frequency was below 5) for categorical variables, the Student’s or Welch’s t test for normally distributed continuous variables, and the Mann-Whitney-U test for not normally distributed continuous variables. We used multivariable logistic regression to analyze the association of sarcopenia with clinical factors, adjusted for age and gender. Pearson’s correlation was used to determine the association between the skeletal muscle areas, indices and radiation attenutation at the different thoracic and lumbar spine levels. Overall and cancer-specific survival was visualized using the Kaplan-Meier-method. Differences in survival curves were assessed with the log-rank test. Multivariable Cox proportional hazards regression determined whether the prevalence of sarcopenia was independently associated with overall and cancer-specific survival. The following covariates were identified and included based on the results of univariable Cox regression, literature^26,27^, clinical experience and general recommendations to avoid overfitting^28^: gender, BMI, age-adjusted CCI, number of resected segments, and pathologic tumor stage. We compared multivariable models where sarcopenia as a dichotomous predictor was substituted by continuous crude muscle measures SMA, SMI and SMRA as well as averaged SMI and SMRA percentiles. Using R percentiles package^29^, the averaged SMI and SMRA percentiles were determined by gender-stratified aggregation of the respective SMI and SMRA percentiles at each available vertebral level in a patient^22^. Model fit was evaluated using Akaike Information Criterion (AIC) and ANOVA testing. 

## Results

### Cohort summary

The study included 280 patients (35.4% female, Table 1) who underwent chest CT. At level T10, due to limited field of view, measurement of skeletal musculature was not possible for three patients who were excluded from the analysis (Fig. 1). 85 (30.4%) patients additionally received a CT scan of the abdomen. And in another 19 (6.8%) thoracic CT examinations, skeletal muscle was completely visualized at level L3. There were no significant sex differences in median age at diagnosis, mean SMRA, ECOG performance status, and comorbid disease burden as per CCI. Compared to men, females had a lower BMI (p < 0.012), SMA (p < 0.001), SMI (p < 0.001), and were less often smokers (p < 0.001). The median age at diagnosis was 66.1 (58.5–72.7) years. 71 (28.0%) of study participants had ECOG 1. Compared to patients with ECOG = 0, patients with ECOG = 1 were significantly older (median age of diagnosis 70.0 vs 63.6 years, p < 0.001) and had a significantly higher burden of comorbidities (median CCI of 1 (0–2) vs 1 (1–3), p = 0.010). Most patients had pathologic tumor stage I (46.9%). The most frequent histology subtypes were adenocarcinoma (46.4%) and squamous cell carcinoma (33.6%). The median time between CT acquisition and lung cancer resection was 22 (6–36) days. Skeletal muscle tissues at level T5, T8 and L3 were completely visualized on the CT scans of 232 (82.9%), 268 (95.7%) and 103 (36.8%) patients, respectively.

**Table 1:** Comparison of patient characteristics between non-sarcopenic and sarcopenic patients.

Variable	All patients (n = 280)	Non-sarcopenic group (n = 185)	Sarcopenic group (n = 95)	P-value
Gender				< 0.001^a^
Female	99 (35.4%)	79 (42.7%)	20 (21.1%)	
Male	181 (64.6%)	106 (57.3%)	75 (79.0%)	
BMI (kg/m^2^)	26.2 (23.2–29.4)	26.6 (23.5–30.0)	24.8 (22.6–27.6)	0.007^c^
BMI WHO category				0.049^a^
Underweight	7 (2.5%)	3 (1.6%)	4 (4.2%)	
Healthy weight	110 (39.3%)	66 (35.7%)	44 (46.3%)	
Overweight	102 (36.4%)	68 (36.8%)	34 (35.8%)	
Obese	61 (21.8%)	48 (25.9%)	13 (13.7%)	
SMRA (HU)				
Female	24.9 (± 7.2)	26.2 (± 7.2)	19.7 (± 4.6)	< 0.001^e^
Male	26.0 (± 7.7)	29.0 (± 7.1)	21.7 (± 6.4)	< 0.001^d^
SMI (cm^2^/m^2^)				
Female	23.1 (20.5–26.6)	23.9 (22.4–27.0)	18.7 (17.0–19.8)	< 0.001^c^
Male	29.1 (25.1–33.2)	32.4 (29.8–35.1)	24.8 (22.9–27.3)	< 0.001^e^
SMA (cm^2^)				
Female	61.7 (± 11.0)	65.0 (± 9.4)	48.4 (± 5.4)	< 0.001^e^
Male	90.1 (± 19.0)	101.0 (± 16.2)	75.1 (± 10.9)	< 0.001^e^
Age at diagnosis (years)	66.1 (58.5–72.7)	65.0 (56.7–72.0)	69.5 (62.0–73.9)	< 0.001^c^
Charlson Comorbidity Index	1 (0–2)	1 (0–2)	2 (1–2)	0.013^c^
Smoking history				0.002^a^
Never	38 (14.0%)	34 (18.9%)	4 (4.4%)	
Ever	233 (86.0%)	146 (81.1%)	87 (95.6%)	
ECOG				0.004^a^
0	183 (72.1%)	132 (78.1%)	51 (60.0%)	
≥ 1	71 (28.0%)	37 (21.9%)	34 (40.0%)	
Largest diameter (cm)	2.7 (1.8–4.5)	2.6 (1.6–4.0)	2.8 (2.0–4.8)	0.136^c^
Histology				0.874^a^
Adenocarcinoma	130 (46.4%)	86 (46.5%)	44 (46.3%)	
Squamous cell carcinoma	94 (33.6%)	63 (34.1%)	31 (32.6%)	
SCLC	11 (3.9%)	6 (3.2%)	5 (5.3%)	
Other NSCLC	45 (16.1%)	30 (16.2%)	15 (15.8%)	
Pathologic tumor stage				0.476^a^
I	130 (46.9%)	89 (48.9%)	41 (43.2%)	
II	49 (17.7%)	27 (14.8%)	22 (23.2%)	
III	68 (24.5%)	45 (24.7%)	23 (24.2%)	
IV	14 (5.1%)	9 (4.9%)	5 (5.3%)	
0	16 (5.8%)	12 (6.6%)	4 (4.2%)	
Neoadjuvant therapy				0.417^a^
No	227 (81.1%)	153 (82.7%)	74 (77.9%)	
Yes	53 (18.9%)	32 (17.3%)	21 (22.1%)	
Adjuvant therapy				0.196^a^
Not recommended	138 (49.3%)	97 (52.4%)	41 (43.2%)	
Applied	114 (40.7%)	73 (39.4%)	41 (43.2%)	
Recommended but unclear if applied	28 (10.0%)	15 (8.1%)	13 (13.7%)	
Procedure				0.581^a^
Minimal-invasive	99 (35.4%)	68 (36.8%)	31 (32.6%)	
Thoracotomy	181 (64.6%)	117 (63.2%)	64 (67.4%)	
Number of resected segments	4 (3–5)	4 (3–5)	4 (3–5)	0.652^c^
Lymphadenectomy				0.261^b^
No	1 (0.4%)	0 (0.0%)	1 (1.1%)	
Partial	6 (2.1%)	3 (1.6%)	3 (3.2%)	
Yes	273 (97.5%)	182 (98.4%)	91 (95.8%)	
VC % predicted	89.6 (± 16.7)	91.7 (± 16.9)	85.5 (± 15.6)	0.004^d^
FEV1% predicted	82.3 (68.4–96.5)	84.5 (71.6–97.8)	78.4 (61.7–93.4)	0.036^c^
DLCO % predicted	58.7 (46.5–75.3)	64.3 (50.2–81.0)	51.4 (43.3–63.5)	< 0.001^c^

Numbers are reported as mean (±SD), median (Q1–Q3) or n (%).

^a^Chi-square test.

^b^Fisher’s exact test.

^c^Mann-Whitney-U test.

^d^Student’s t test.

^e^Welch’s t test.

### Sarcopenia 

**Figure 2:**
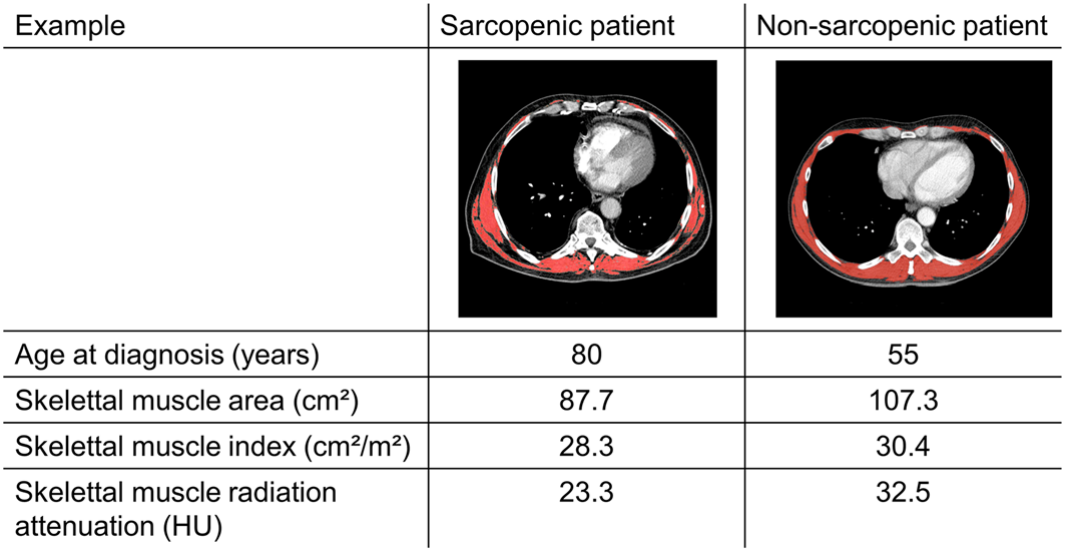
Comparison of skeletal muscle area, index and radiation attenuation between sarcopenic and non-sarcopenic male patients. First row: pixels identified as muscle (red) superimposed on axial computed tomography images at the level of the tenth thoracic vertebral body.

95 (33.9%) patients were classified sarcopenic (Table 1). Of note, the prevalence of sarcopenia was 20.2% in females and 41.4% in males. Figure 2 shows comparison of a male sarcopenic to a male non-sarcopenic patient. Compared to non-sarcopenic patients, sarcopenic patients were older (median age of diagnosis 69.5 vs 65.0 years, p < 0.001), had more comorbidities according to the CCI (median of 2 (1–2) vs 1 (0–2), p = 0.013) and more frequently an impaired ECOG performance status of 1 (frequency of 40.0% vs 21.9%, p = 0.004). After adjusting for age and gender, sarcopenia was significantly associated with lower BMI (OR = 0.88, p < 0.001), higher frequency of positive smoking history (OR = 5.05, p = 0.005) and a lower preoperative DLCO % predicted (OR = 0.97, p < 0.001). Other lung function parameters, FEV1% predicted (OR = 0.99, p = 0.122) and VC % predicted (OR = 0.99, p = 0.113) did not reach the significance level. There were no significant differences between the sarcopenic and non-sarcopenic group with respect to histologic subtypes, pathologic tumor stage, neoadjuvant and adjuvant therapy, procedure, and number of resected segments.

**Figure 3:**
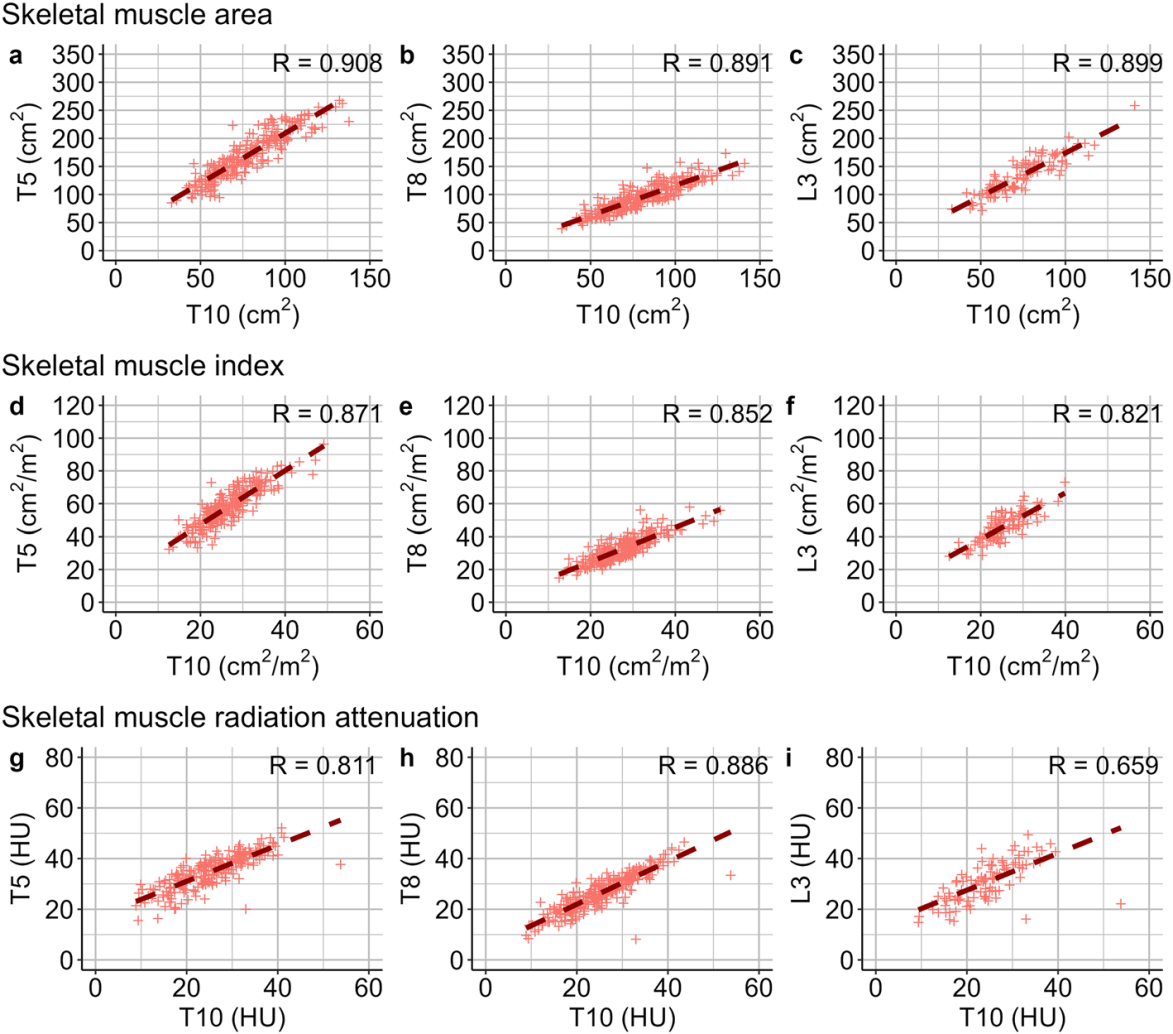
Correlation analysis of skeletal muscle areas (SMA; **a–c**), indices (SMI; **d–f**), and radiation attenuation (SMRA; **g–i**). SMA, SMI and SMRA at the level of the tenth (T10) thoracic vertebral body were compared to SMAs, SMIs and SMRAs at the level of the fifth (T5) and eighth (T8) thoracic and third (L3) lumbar vertebral body, respectively. Pearson’s Rho ranges from 0.659 to 0.908. P < 0.001 for all.

T10-SMA correlated highly with SMA at level T5, T8 and L3 (Pearson’s Rho ≥ 0.891 for all, p < 0.001 for all, Fig. 3). Similarly, Pearson’s correlation coefficients for the correlation of T10-SMI with SMI at level T5, T8 and L3 were ≥ 0.821 (p < 0.001 for all), respectively. SMRA at level T10 correlated well with SMRA at level T5, T8 and L3 (Pearson’s Rho ≥ 0.659 for all, p < 0.001 for all).

### Survival

**Figure 4:**
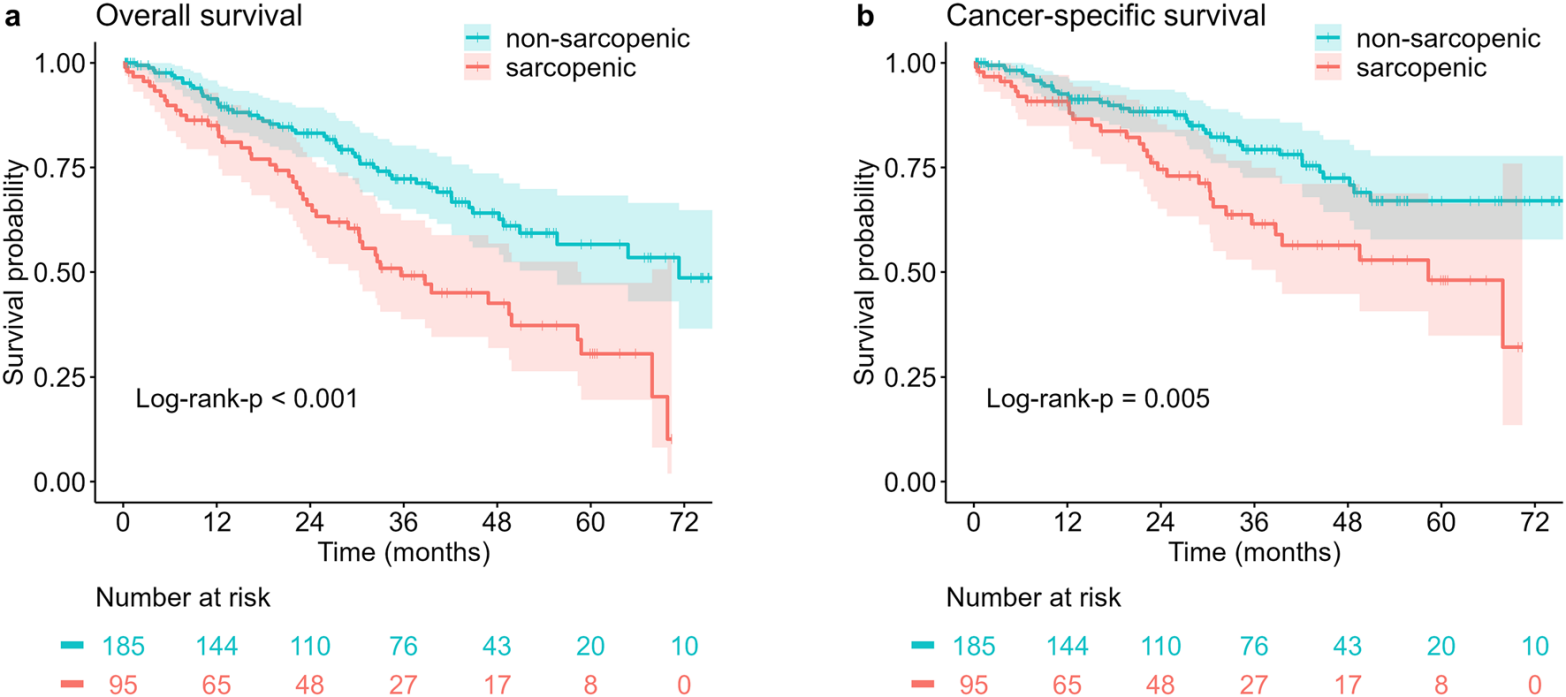
Kaplan-Meier analysis of overall (**a**) and cancer-specific survival (**b**) of patients with lung cancer following anatomic resection. Survival and number at risk of the sarcopenic (red) and non-sarcopenic group (blue) are plotted in monthly intervals. Red and blue areas indicate 95%-confidence intervals. Log-rank-p < 0.001 for overall and 0.005 for cancer-specific survival.

During a median follow-up period of 55.7 (46.8–71.3) months, death of 100 (35.7%) patients was registered. 53 (28.6%) of non-sarcopenic patients died, whereas 47 (49.5%) of sarcopenic patients died. In the Kaplan-Meier analysis, the sarcopenic group had worse overall (log-rank-p < 0.001) and cancer-specific survival (log-rank-p = 0.005, Fig. 4). There was no prognostic difference for overall and cancer-specific survival between the two most common histologic subtypes adenocarcinoma and squamous cell carcinoma (Table 2 and 3). Therefore, we did not add histology as a covariate to the multivariable model.

After multivariable adjustment, sarcopenia was independently associated with worse overall (hazard ratio (HR) 2.11, 95%-confidence interval (95%-CI) 1.38–3.23, p < 0.001, Table 2) and cancer-specific survival (HR 2.00, 95%-CI 1.19–3.36, p = 0.009, Table 3). In addition, pathologic tumor stage was found to be an unfavorable prognostic factor for overall survival as well as for cancer-specific survival. This multivariable Cox regression model using presence of sarcopenia as a binary variable provided a significantly better model fit as per AIC compared to models comprising continuous SMA, SMI and SMRA and averaged SMI percentiles as predictors for overall survival (p < 0.001, Table 4). Only the model that included averaged SMRA percentiles showed a lower AIC value in comparison to the use of dichotomous sarcopenia predictor.

**Table 2:** Univariable and multivariable Cox proportional hazards regression for overall survival of the study cohort.

Variable	Univariable HR (95%-CI)	P-value	Multivariable HR (95%-CI)	P-value
Sarcopenia				
No	Ref			
Yes	2.14 (1.44 –3.18)	< 0.001	2.11 (1.38–3.23)	< 0.001
Gender				
Female	Ref			
Male	1.24 (0.81–1.88)	0.325	1.02 (0.65–1.61)	0.928
BMI (kg/m^2^)	0.98 (0.94–1.02)	0.221	0.98 (0.94–1.03)	0.412
Age-adjusted Charlson Comorbidity Index	1.06 (0.98–1.15)	0.159	1.08 (0.99–1.19)	0.097
Number of resected segments	1.08 (0.98–1.18)	0.132	1.05 (0.95–1.16)	0.357
Pathologic tumor stage				
I	Ref			
II	1.90 (1.07–3.35)	0.028	1.72 (0.97–3.06)	0.063
III	1.78 (1.09–2.92)	0.022	1.80 (1.06–3.06)	0.029
IV	3.94 (1.92–8.09)	< 0.001	5.05 (2.36–10.81)	< 0.001
0	1.68 (0.70–4.04)	0.249	1.97 (0.80–4.87)	0.142
Histoloy				
Adenocarcinoma	Ref			
Squamous cell carcinoma	1.09 (0.71–1.68)	0.697		
SCLC	2.19 (0.93–5.15)	0.073		
Other NSCLC	0.68 (0.34–1.34)	0.264		
FEV1% predicted (per 10%-increment)	0.97 (0.87–1.07)	0.52		

Numbers are reported as median (Q1–Q3).

**Table 3:** Univariable and multivariable Cox proportional hazards regression for cancer-specific survival of the study cohort.

Variable	Univariable HR (95%-CI)	P-value	Multivariable HR (95%-CI)	P-value
Sarcopenia				
No	Ref			
Yes	1.93 (1.17–3.20)	0.010	2.00 (1.19–3.36)	0.009
Gender				
Female	Ref			
Male	1.27 (0.74–2.18)	0.395	1.08 (0.63–1.87)	0.777
BMI (kg/m^2^)	0.99 (0.95–1.04)	0.816	0.99 (0.94–1.04)	0.751
Age-adjusted Charlson Comorbidity Index	1.01 (0.90–1.13)	0.901	1.03 (0.91–1.16)	0.672
Number of resected segments	1.08 (0.96–1.22)	0.197	1.04 (0.92–1.18)	0.533
Pathologic tumor stage				
I	Ref			
II	2.76 (1.36–5.58)	0.005	2.27 (1.14–4.51)	0.019
III	1.95 (1.00–3.81)	0.049	1.93 (0.99–3.76)	0.052
IV	5.56(2.37–13.02)	< 0.001	6.53 (2.77–15.36)	< 0.001
0	1.54 (0.45–5.29)	0.495	1.50 (0.43–5.23)	0.523
Histoloy				
Adenocarcinoma	Ref			
Squamous cell carcinoma	1.30 (0.75–2.24)	0.352		
SCLC	3.53 (1.45–8.61)	0.005		
Other NSCLC	0.35 (0.10–1.14)	0.081		
FEV1% predicted (per 10%-increment)	1.00 (0.87–1.14)	0.946		

Numbers are reported as median (Q1–Q3).

**Table 4:** Comparison of different multivariable Cox proportional hazards regression models for overall survival.

Predictor	AIC	P-value
Sarcopenia (ref.)	955.1	Ref
SMA (cm^2^)	957.6	< 0.001
SMI (cm^2^/m^2^)	958.9	< 0.001
SMRA (HU)	955.8	< 0.001
Averaged SMI percentile	957.2	< 0.001
Averaged SMRA percentile	951.6*	< 0.001

Multivariable regression models included the predictor indicated in the first column in addition to the following covariates: gender, body mass index, age-adjusted Charlson Comorbidity Index, number of resected segments, and pathologic tumor stage. Models were compared to the reference model using ANOVA, respectively.

*AIC* Akaike Information Criterion.

## Discussion

While CT imaging at the third lumbar vertebra is considered gold standard to quantify skeletal musculature, it is, however, not readily available in some patient groups, including lung cancer patients. This study evaluated a method to diagnose sarcopenia on chest CT in accordance with the widely accepted European consensus diagnostic criteria and cutoff recommendations^1,13^. At the tenth thoracic vertebral level, sarcopenia was indicated by low skeletal mass index in conjunction with low skeletal muscle radiation attenuation to account for presence of all diagnostic criteria—muscle quantity, quality, strength, and physical performance^1^—with reference to previously published cutoff values^1,13^. Sarcopenia was an independent prognostic factor for poorer long-term overall and cancer-specific survival of lung cancer patients following anatomic resection with curative intent. In addition, T10-SMI and SMRA correlated well with muscle indices and densities at other thoracic and lumbar spine levels, respectively. Our study emphasizes that assessment of sarcopenia solely by chest CT is feasible, easily translatable into routine clinical practice, and helpful to improve preoperative risk stratification of lung cancer patients undergoing surgery. 

In our study, presence of sarcopenia evaluated by SMI and SMRA on thoracic CT was independently associated with worse overall and cancer-specific survival of lung cancer patients scheduled for anatomic resection. This observation held true after adjusting for variables known to be associated with mortality following lung cancer surgery^26,27^. Additionally, pathologic tumor stage was found to be an independent predictor of survival. In otherwise identical models, including sarcopenia as a dichotomous predictor for overall survival resulted in a model with significant higher goodness of fit compared to the use of singular continuous muscle measures SMA, SMI and SMRA as well as averaged SMI percentiles that aggregated skeletal muscle indices from all available measurement levels in a single patient. Still, averaged multi-vertebral level SMRA percentiles yielded a better fitted model, underlining the additional value of muscle quality assessment to determine sarcopenia. 

SMRA relates to muscle quality^1,6,7^ and is associated with function^7–10^, strength^7,11,12^ and physical performance^13^. Hence, it can bypass unavailability of functional testing^13^. All diagnostic criteria for sarcopenia recommended by European and Asian consensus can be met by SMRA in conjunction with a parameter for muscle quantity, such as SMA or SMI, and, enable diagnosis of sarcopenia solely by chest CT if cutoff values are specified. The European consensus recommended to set cutoffs two standard deviations below the mean of a young, healthy reference population^1^. In accordance with this guideline, reference values for SMI and SMRA derived from kidney donors aged 18–40 years have been published for levels T10 to L3^13^. Hence, our study highlights feasibility of sarcopenia assessment based on a single modality that is readily available in lung cancer patients. The preoperative staging of lung cancer includes a contrast-enhanced CT scan of the chest and upper abdomen that usually does not encompass L3^16^, the gold standard vertebral level for sarcopenia assessment^1^. Consistently, only 36.8% of patients underwent additional CT of the abdomen and 6.8% of chest CT scans also included level L3. Nevertheless, our results showed that measures of skeletal muscle quantity, SMA and SMI, as well as quality, SMRA, at level L3 correlate well with measures at level T10, the most cranial thoracic spine level for which reference values from a young, healthy reference population have previously been published^13^. In addition, T10-SMA, SMI and SMRA correlated well with SMAs, SMIs and SMRAs of the upper and middle thoracic spine. Compared to level T5 and T8, skeletal musculature was more frequently completely visualized at level T10. This finding is consistent with other study groups^22,23,30^. 

Consistent with the majority of studies, a higher percentage of men were affected by sarcopenia (41.4% vs 20.2%)^31–33^. Compared to non-sarcopenic patients, sarcopenic patients were significantly older, had a higher ECOG and burden of comorbid disease as per CCI. After adjustment for age and gender, sarcopenia was significantly associated with a lower BMI and lower preoperative DLCO % predicted. Hence, our findings are consistent with the pathophysiology of sarcopenia^3^. With a median age at diagnosis of 69.5 years, the sarcopenic patients were at a stage in life with ongoing, physiologic decline in skeletal muscle mass and maximization of body fat^12,34^. The western lifestyle, especially excessive energy intake, sedentary behavior, and physical inactivity, contribute to fat deposition in muscle and muscle loss^35,36^. Chronic diseases such as chronic obstructive pulmonary disease, type 2 diabetes mellitus and inflammatory processes promote muscle mass reduction^3^. Furthermore, malignancies can lead to cancer cachexia including muscle wasting and physical impairment^37^ that may become apparent as a higher ECOG and poorer preoperative lung function. Yet, sarcopenia can be therapeutically targeted by aerobic and resistance training^38,39^, protein supplement^40^ and Mediterranean diet^41^. Thus, intervention may improve sarcopenia and possibly clinical outcomes. 

To date, the European consensus criteria for sarcopenia which closely align with the Asian consensus are most widely recognized for clinical practice and research^1–3^. We have outlined that all criteria of the European consensus can be met radiologically by CT-derived SMI and SMRA^1^. To facilitate implementation in daily clinical practice, additional tests may be omitted^13^. So far, lumbar assessment of skeletal muscle is considered the gold standard to quantify sarcopenia^1^. Recent availability of cutoff values from a young, healthy reference population, allows the diagnosis of sarcopenia on chest CT which—unlike CT imaging of the abdomen—is readily available in lung cancer patients^13^. This is the first time these thoracic reference values have been tested against clinical outcomes. Our retrospective analysis showed that, in a first step, the skeletal muscle parameters SMA, SMI and SMRA at the third lumbar vertebra level correlated well with those at the tenth thoracic vertebra level. In a second step, preoperative sarcopenia as determined by SMI and SMRA at level T10 was shown to be an independent prognostic factor for poor overall and cancer-specific long-term survival of lung cancer patients following anatomic resection. Thus, our study implements single modality-based evaluation of sarcopenia on chest CT in accordance with European and Asian consensus guidelines, demonstrating its feasibility for straightforward integration into routine clinical practice and its value in enhancing preoperative risk stratification of lung cancer patients scheduled for surgery.

There are several limitations to our study. First, the retrospective design resulted in a 47%-exclusion rate due to unavailable imaging or clinical data elements. Furthermore, we had to retrospectively convert some TNM stages from the seventh to the eighth edition. Third, no minimum was set for the follow-up interval. However, the median follow-up period was 55.7 months. Fourth, the standard of care staging protocol for lung cancer patients includes IV contrast-enhanced CT imaging of the chest and upper abdomen. Consequently, most of the CT scans were acquired with contrast-enhancement and, for standardization purposes, patients who underwent plain CT imaging were excluded from the study. The cutoff-values used for sarcopenia diagnosis, however, originated from non-contrast-enhanced CT imaging at 120kVp^13^. It was shown that contrast injection can increase radiation attenuation by up to 5.99%^42^. Conversely, increase in slice thickness may decrease radiation attenuation^42^. Since this study included CT scans with a slice thickness of 3 to 5 mm, this may affect threshold-based segmentation. Overall, IV contrast, slice thickness, tube voltage and current may cause slight variations of skeletal muscle measures^42^. Nevertheless, sarcopenia was defined by both low SMRA and SMA normalized by patients’ height, and influence of IV contrast on SMA was shown to be minimal (up to 1.88%^42^). While, we used level T10 since it was the most cranial level for which cutoff-values from a young, healthy reference population were available, other thoracic levels may also be utilized. Finally, only patients that deemed fit enough for anatomic resection, were included in this study. Therefore, results may not be generalizable to patients who were assigned to other treatment modalities.

Future longitudinal observational studies that repeatedly assess sarcopenia-related measures in the pre- and postoperative course may yield important insights into the dynamics of muscle wasting and guide implementation of targeted interventions. Furthermore, conversion of the semi-automated sarcopenia quantification into a fully automated pipeline may allow immediate detection of sarcopenia when CT scans for diagnosis and staging purposes are performed as well as direct inclusion in preoperative risk stratification models.

## Data Availability

The datasets generated and/or analyzed during the current study are available from the corresponding author upon reasonable request.
